# Functional Evaluation of AMD-Associated Risk Variants of Complement Factor B

**DOI:** 10.1167/iovs.61.5.19

**Published:** 2020-05-14

**Authors:** Camilla Pilotti, John Greenwood, Stephen E. Moss

**Affiliations:** UCL Institute of Ophthalmology, London, United Kingdom

**Keywords:** retina, complement, angiogenesis, AMD, CFB

## Abstract

**Purpose:**

The 32W and 32Q variants of complement factor B (CFB) are associated with reduced risk of developing neovascular age-related macular degeneration (AMD) compared with the common 32R allele. The objective of this study was to determine if the most protective R32Q variant affects the neovascular process in a manner consistent with the reported reduced disease association.

**Methods:**

The 32R, 32W, and 32Q human CFB variants were expressed in human embryonic kidney 293T cells and purified from culture supernatant. The ex vivo mouse fetal metatarsal explant model was used to investigate the effect of these three human CFB variants on angiogenesis. Metatarsal bones were isolated from mouse embryos and cultured in the presence of the three CFB variants, and angiogenesis was measured following immunostaining of fixed samples. ELISAs were used to quantify C3 and VEGF protein levels in metatarsal culture and quantitative PCR to measure *Cfb*, *C3*, and *Vegf* expression.

**Results:**

We show here that the three CFB variants have different biological activities in the mouse metatarsal assay, with CFB^R32^ exhibiting significantly greater angiogenic activity than CFB^Q32^ or CFB^W32^, which were broadly similar. We also observed differences in macrophage phenotype with these two variants that may contribute to their activities in this experimental model.

**Conclusions:**

We have demonstrated that the biological activities of CFB^R32^, CFB^W32^, and CFB^Q32^ are consistent with their AMD risk association, and we provide functional evidence of roles for these variants in angiogenesis that may be relevant to the pathogenesis of the neovascular form of AMD.

The neovascular form of age-related macular degeneration (AMD) is the leading cause of registered blindness worldwide.^1^ Choroidal neovascularization (CNV) is the principal pathologic feature of this form of AMD and involves the growth of new blood vessels in the subretinal tissue, causing loss of central vision.^2^ AMD is a multifactorial and complex disease in which the molecular pathogenesis has not yet been fully elucidated. However, dysregulation of the complement system, associated with a number of well-defined single nucleotide polymorphisms (SNPs) in genes of the alternative pathway (AP) of complement activation, has a contributory role in many patients with AMD. Although haplotypes in complement factor H are the most common in AMD, genetic studies have revealed significant associations between SNPs in several complement genes, including complement factor B (CFB), and AMD risk.^3–7^

CFB is mainly produced by hepatocytes but is also expressed in the choroid plexus in humans (EMBL-EBI database), in the mouse retina, and at low levels in retinal pigment epithelial (RPE) cells.^8^ CFB is a fluid-phase positive regulator of the AP, with its main activity being driven by the Bb subunit that binds C3b, the activated portion of C3. The C3bBb complex in turn drives the formation of the C3 convertase, the downstream C5 convertase, and generation of the membrane attack complex.

The H1 haplotype, common to both the CFB and complement C2 genes, has been shown to be associated with a significantly higher risk of developing AMD. However, in contrast to most genetic associations of complement genes with AMD, the L9H and the R32Q variants of CFB are protective against the neovascular form of AMD.^4^ Furthermore, it has been demonstrated that the R32W variant of CFB has an intermediate protective effect against neovascular AMD compared to R32Q.^9^ The greater protection afforded by CFB^Q32^ correlates with its decreased hemolytic activity, which is explained by its reduced ability to bind C3b and to form the proenzyme C3bB; therefore, activation of the AP is less efficient.^6^ CFB variants distinct to those associated with AMD risk were also found to be associated with retinopathy of prematurity, which involves abnormal neovascularization in the retinas of preterm babies.^10^ Collectively, these reports lend weight to the idea that CFB may be particularly associated with the vascular complications of retinal disease.

Animal models of retinal vascular disease also support a role for CFB in the development of neovascularization in the eye. Thus, CFB is upregulated in CNV, and the AP was shown to be involved in the development of CNV in the mouse model of laser-induced CNV, as gene knockout of *Cfb* led to reduced pathologic ocular angiogenesis compared to wild type and to mice with a compromised classical or lectin pathway.^11^ In addition, AP activation was shown to be necessary, but not alone sufficient, for the development of laser-induced CNV because mice with a functional AP, but no classical and lectin pathway (C1q^−/−^ MBL^−/−^), developed similar lesion sizes to CFB knockout mice.^12^ Studies using transgenic mice expressing CFB only in the RPE-choroid (CFB-tg) demonstrated that local production of CFB in the eye is sufficient to activate complement leading to vascular pathology.^13^ In this study, we use an ex vivo explant model to ask whether the common and low-risk AMD variants of CFB directly affect angiogenesis and, if so, whether their biological activities are consistent with their observed association with AMD risk. Our data suggest that CFB may indeed play a role in vascular pathology in the eye with the R32 variant of CFB having greater angiogenic activity.

## Materials and Methods

### Animals

C75Bl/6J mice were purchased from Charles River Laboratories, Écully, France and bred in-house. All procedures were performed in accordance with the UK Animals (Scientific Procedures) Act and with the Association for Research in Vision and Ophthalmology Statement for the Use of Animals in Ophthalmic and Vision Research, as well as the Animal Welfare and the Ethical Review Bodies of the UCL Institute of Ophthalmology.

### Metatarsal Angiogenesis Assay

The metatarsal angiogenesis assay was performed as described^14^ with minor changes. Metatarsal bones were exposed to various treatments, including 10% fetal bovine serum, PBS, 10% heat-inactivated human serum, 10% human serum, human CFB^R32^ (200 μg/mL), and CFB^Q32^ (200 μg/mL). The concentration of CFB used in these studies is in the physiologic range of CFB in human serum.^13^ At day 11 of culture, conditioned media were collected for analysis, the metatarsals were fixed in 4% Paraformaldehyde (PFA), permeabilized in 10% BSA with 0.1% Triton, and stained overnight at 4°C for CD31 (553370; BD Pharmingen/BD Biosciences, CA, USA), complement C3 (55500; Cappel/MP Biomedicals, Cambridge, UK), F4/80 (MCA497R; AbD Serotec, Kidlington, UK), and Arginase-1 (SC-18351; Santa Cruz, CA, USA). The secondary antibodies were incubated for 2 hours at room temperature and samples were imaged. After image processing in ImageJ (National Institutes of Health, Bethesda, MD, USA) to mask the cartilage, the total length of CD31-positive tubular structures and the number of junctions were quantified by Angiosys (TCS Cellworks, Buckingham, UK) using manual thresholding. The area of staining was quantified using ImageJ, statistical analysis was performed using GraphPad Prism (6.01; GraphPad Software, La Jolla, CA, USA), and one-way ANOVA was used to determine statistical significance between test groups. For confocal imaging, metatarsals were cultured as described^14^ but on coverslips, then mounted on glass slides and imaged using maximum-intensity projections (ZEISS LSM 700 confocal microscope; ZEISS, Cambridge, UK).

### ELISA

ELISA was used to measure C3 and VEGF in the conditioned media collected from the metatarsal assays. For the C3 ELISA, plates were precoated overnight at 4°C with polyclonal goat IgG to mouse complement C3 (55463, 1:8000; MP Biomedicals, Cambridge, UK). Plates were washed with 0.2% Tween in PBS and blocked with 2% BSA and 0.2% Tween for 1 hour at room temperature. After washing, conditioned media from metatarsals and standards (normal human serum with known concentration of C3 protein^15^) were added for 1 hour at room temperature in blocking buffer in 12 serial 2× dilutions (1:1000 to 1:2,048,000). Standards ranged from 0.1 to 220 ng/mL and the experimental readings were between 0 and 27 ng/mL. All samples were analyzed in duplicate. After washing, horseradish peroxidase (HRP)–conjugated goat anti-mouse C3 (55557, 1/25,000; MP Biomedicals) was added for 1 hour at room temperature. After washing, HRP Substrate Reagent (DY993; R&D, Systems, Minneapolis, Minnesota, USA) and stopping solution (2N sulfuric acid, 895032; R&D) were added and the optical density was measured at 450 nm, with 540 nm set as the reference. Data were analyzed with GraphPad Prism (6.01) using nonlinear regression analysis, with a dose-response equation by interpolating unknown values from a standard curve of known values. For VEGF, the DuoSet ELISA kit for mouse VEGF (DY493-05; R&D) was used following the manufacturer's instructions.

### Quantitative PCR

RNA from metatarsal cultures was extracted (74106; QIAGEN, Hilden, Germany) 24 hours after the final treatment and transcribed into cDNA (205310; QIAGEN) following the manufacturer's instructions. Quantitative PCR reactions were performed using PCR master mix (4367659; ThermoFisher Scientific, Loughborough, UK) and relative gene expression was determined using the ΔCt method. Results were normalized to the housekeeping gene and to PBS for each experiment. Primer sequences used were as follows: *C3*, forward primer 5′-AGACACAAAGGACCTGGAACTGCT-3′, reverse primer 5′-AGGCAGTCTTCTTCGGTGTGTGAA-3′; *Cfb*, forward primer 5′-TACCCCGTGCAGACTCGAA-3′, reverse primer 5′-GTGGGCAGCGTATTGCTCT-3′; *Vegf*, forward primer 5′-GACTTGTGTTGGGAGGAGGA-3′, reverse primer 5′-TCTGGAAGTGAGCCAATGTG-3′; and *β-tubulin*, forward primer 5′-CAAAGTGTCTGATACCGTGGTC-3′, reverse primer 5′-GCTTGAGGGTACGGAAGCA-3′.

### Cloning of CFB Variants

The PCR fragment CFB-Hisx6 (I.M.A.G.E clone, ID: 2959706/AU29 G01 M13F; Source Bioscience, Nottingham, UK) containing the DNA sequence of the W32 variant of human CFB was ligated into the mammalian expression vector pcDNA3.1 (Invitrogen, Loughborough, UK) at the AflII/ApaI site (forward primer: GTCTCAGCTGCTTAAGGTCCGTATGGGGAGCAATCTCAGC, reverse primer: TACTATATCGGGGCCCCA TTCTAGTGATGATGATGGTGATGTAGAAAACCCAAATCCTCATC). Site-directed mutagenesis (210518; Agilent Technologies, CA, USA) was used to obtain the R32 and Q32 variants: hCFB^R32^ and hCFB^Q32^ (Q32 forward primer: CAGGATCCCTGGGGCTGGGCCAAAGACCATGG, reverse primer: CCATGGTCTTTGG CCCAGTTTTAGGGATCCTG; R32 forward primer: GATCCCTGGGGCCGGGCCAAAGACCAT, reverse primer: ATGGTCTTTGGCCCGGCCCCAGGGATC). All DNA sequences were verified by Sanger sequencing.

### CFB Expression in Mammalian Cells

The pcDNA3.1-CFB expression vectors were stably expressed in human embryonic kidney (HEK) 293T cells (R790-07; Invitrogen) maintained in freestyle expression medium supplemented with Glutamax. Cultures were grown to confluency at 37°C in a humidified atmosphere of 8% CO_2_ on an orbital shaker. Transfections were carried out using 40 μg of each plasmid, Opti-MEM (31985-070; Life Technologies, Loughborough, UK) and Lipofectamine 2000 (11668019; Life Technologies). Forty-eight hours following transfection, cells were resuspended in medium containing 0.2 mg/mL G418 (Sigma, Dorset, UK) to select for transfected cells only. Three weeks after transfection, supernatants containing the secreted recombinant Human Complement Factor B (hCFB) proteins were collected.

### Recombinant CFB Purification

The His-tagged hCFB protein variants were purified using the ÄKTA pure 150 Protein Purification System (GE Healthcare Life Sciences, Buckinghamshire, UK) via affinity chromatography. The HisPrep FF16/10 column (total column volume [VT]: 20 mL; GE Healthcare Life Sciences) was used in conjunction with His Buffer Kit (GE Healthcare Life Sciences) according to the manufacturer's instructions with minor changes. The purified protein was buffer exchanged into HEPES-buffered saline using the HiPrep 26/10 Desalting column (void volume [V0]: 15 mL and VT is 53 mL; GE Healthcare). All the proteins used in this study had endotoxin levels below 2 EU/mL (PSD250-30; Cape Cod, Massachusetts, USA) at the working concentration.

### Protein Detection and Analysis Techniques

Purified proteins were resolved by SDS-PAGE and gels stained with Coomassie blue. NuPAGE Novex precast 4% to 12% Bis-Tris gels (NP0321BOX; ThermoFisher Scientific) were run in NuPAGE MOPS (3-(N-Morpholino) propanesulfonic acid) SDS running buffer (NP0001-02; ThermoFisher Scientific). NuPAGE lithium dodecyl sulfate sample buffer (Life Technologies, NP0008, with 100 mM DL-dithiothreitol; ThermoFisher Scientific, R0861) was added to the samples before heat denaturation for 5 minutes at 95°C. Gels were run at 150 V and 400 mA for 90 minutes, then fixed in 25% isopropanol and 10% acetic acid in water for 30 minutes, as well as stained in 10% acetic acid in double-distilled water containing Coomassie Brilliant Blue R 250 (27816; Sigma) at 60 mg/L.

## Results

### Generation of Recombinant Human CFB Variants

**Figure 1.**
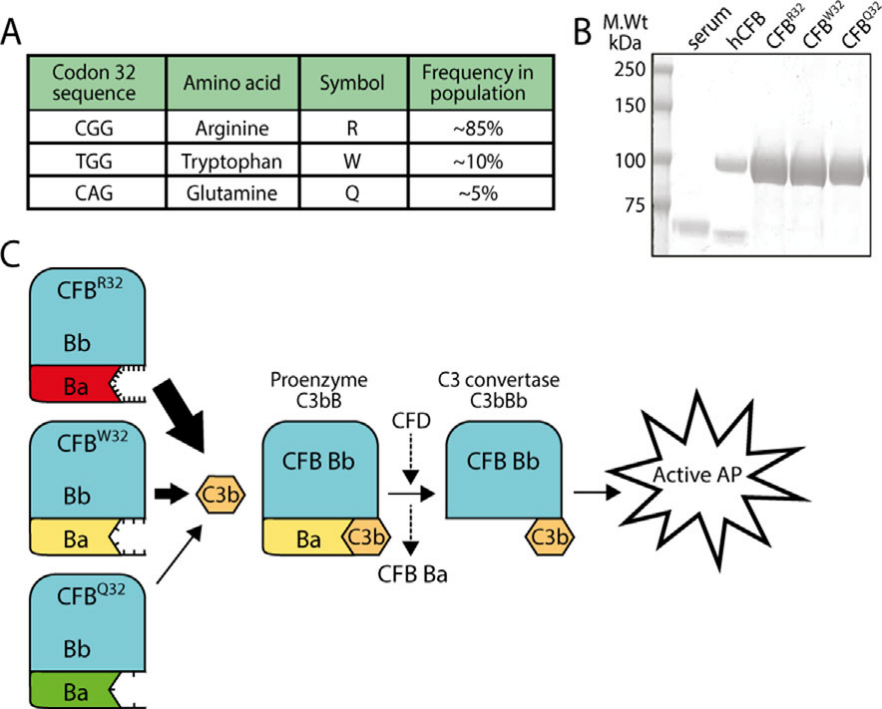
Generation of human recombinant CFB variants and their role within the alternative pathway. (**A**) Table of the most common CFB polymorphisms, their sequences at codon 32, and the allelic frequencies in the population (adapted from Hughes et al.^9^). (**B**) Coomassie-stained SDS-PAGE gel showing protein molecular weight markers, then from left to right 1:100 whole human serum (the band at ∼68 kDa is assumed to be albumin), 1 µg commercial hCFB, 10 µg each of purified hCFB^R32^, hCFB^W32^, and hCFB^Q32^. (**C**) Polymorphisms in the Ba domain of hCFB affect binding affinity to C3b and cause different levels of complement activation that correlate with AMD risk. When the Ba domain carries the R32 polymorphism, C3b binds to it, strongly leading to efficient proenzyme C3bB formation, and CFD then drives the proteolytic cleavage of the Ba domain to form the C3 convertase C3bBb. The more C3 convertase molecules are formed, the more complement is amplified. In contrast, when the Ba domain carries the Q32 polymorphism, C3b binds to it weakly, leading to less proenzyme C3bB and C3 convertase formation and therefore reduced complement activation. The W32 variant exhibits intermediate activity.

Following the discovery of a significant association between human CFB variants (CFB^Q32^ and CFB^W32^) and a reduced risk of developing neovascular AMD,^4^^,^^9^ we wanted to generate the recombinant proteins to test whether they have any influence on angiogenesis when compared to the most common variant (CFB^R32^).  Figure 1A shows the codon of interest of the three proteins, the single amino acid change (R, W, or Q) and their allelic frequencies in the population, with CFB^R32^ being the most common.

Following expression in HEK cells, the secreted recombinant hCFB proteins were visualized on SDS-PAGE gels to confirm their identity, purity, and concentration (Fig. 1B). A single polypeptide band of the expected size was observed in each case. Figure 1C shows in schematic form the relative biological activities of the three variants: CFB^R32^ has the highest binding affinity to C3b and therefore activates complement with the greatest efficiency, and CFB^Q32^ binds C3b with lower affinity and thus complement activity is reduced. CFB^W32^ exhibits an intermediate level of complement activation.

### CFB and Angiogenesis

**Figure 2.**
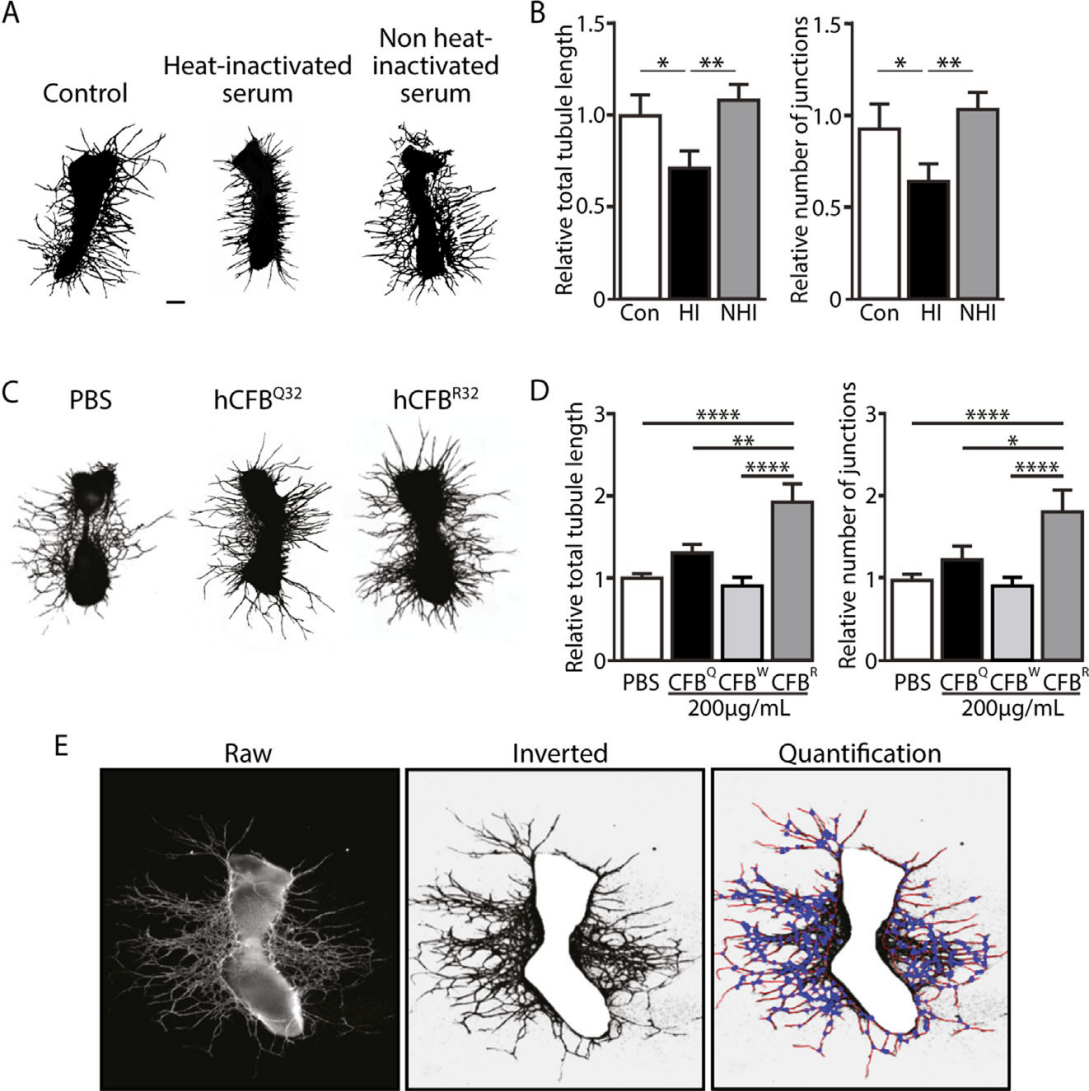
Impact of complement proteins on angiogenesis. (**A**) Representative images of metatarsals stained with CD31 treated with normal medium, 10% HI, and 10% NHI. *Scale bar*: 200 µm. (**B**) Quantification of sprouting expressed as total tubule length in metatarsals treated with HI and NHI and quantification of branching expressed as number of junctions in metatarsals treated with normal medium, HI, and NHI. (**C**) Representative images of CD31-stained metatarsals treated with medium supplemented with PBS, 200 µg/mL CFB^Q32^ and CFB^R32^. Note that CFB^W32^ resembled CFB^Q32^ and is not shown. *Scale bar*: 200 µm. (**D**) Quantitation of sprouting, expressed as total tubule length, and quantitation of branching, expressed as number of junctions in PBS-treated metatarsals compared to 200 µg/mL CFB^Q32^-, CFB^W32^-, and CFB^R32^-treated metatarsals. (**E**) Processing and quantitation of metatarsal images. The raw image has a *black* background and the CD31-positive pixels are *white* (vessels and bone); the colors are then inverted to render the vessels *black* and the background *white*, and the bone is masked and removed manually. Finally, the image is quantified automatically using Angiosys with tubules shown in *red* and vessel branch points in *blue*. Data are normalized to the relative controls. *Error bar*s: SEM. *P < 0.05, **P ≤ 0.01, ****P ≤ 0.001.

To investigate whether the CFB^Q32^ and/or CFB^W32^ variants have a modulatory role in angiogenesis, the ex vivo mouse metatarsal model was used. First, we examined 11-day metatarsals treated with 10% heat-inactivated fetal bovine serum (control), as well as 10% heat-inactivated (HI) and non-heat-inactivated (NHI) human serum and stained for CD31 (Fig. 2A). Vessel outgrowth and branching were both increased in NHI-serum-treated metatarsals compared to HI-serum-treated metatarsals (Fig. 2B). The positive impact of human serum on murine angiogenic sprouting and branching provides evidence of the functional activity of the human proteins in a mouse system, and the reduced activity of the HI-serum shows that heat-labile serum components, such as complement proteins, contribute to the observed angiogenic growth.

Because CFB is a key complement activator, we next examined the impact of CFB^Q32^, CFB^W32^, and the commoner CFB^R32^ variant on angiogenesis in the metatarsal assay. Mouse metatarsals (E17.5) were prepared using tissues from wild-type C57/Bl6; treated with 200 µg/mL CFB^Q32^, CFB^W32^, or CFB^R32^; and stained for CD31 and imaged at 11 days postplating (Fig. 2C). Vessel outgrowth and branching from explanted metatarsals were analyzed following image analysis (Fig. 2E), which revealed a significant increase in the presence of exogenous CFB^R32^. In contrast, no significant impact on angiogenesis was observed when metatarsals were cultured with the two protective CFB variants (Fig. 2D). Note that because the CFB^W32^ variant showed equivalent activity to CFB^Q32^ in these assays, we did not pursue it further.

### CFB and Complement Activation

**Figure 3.**
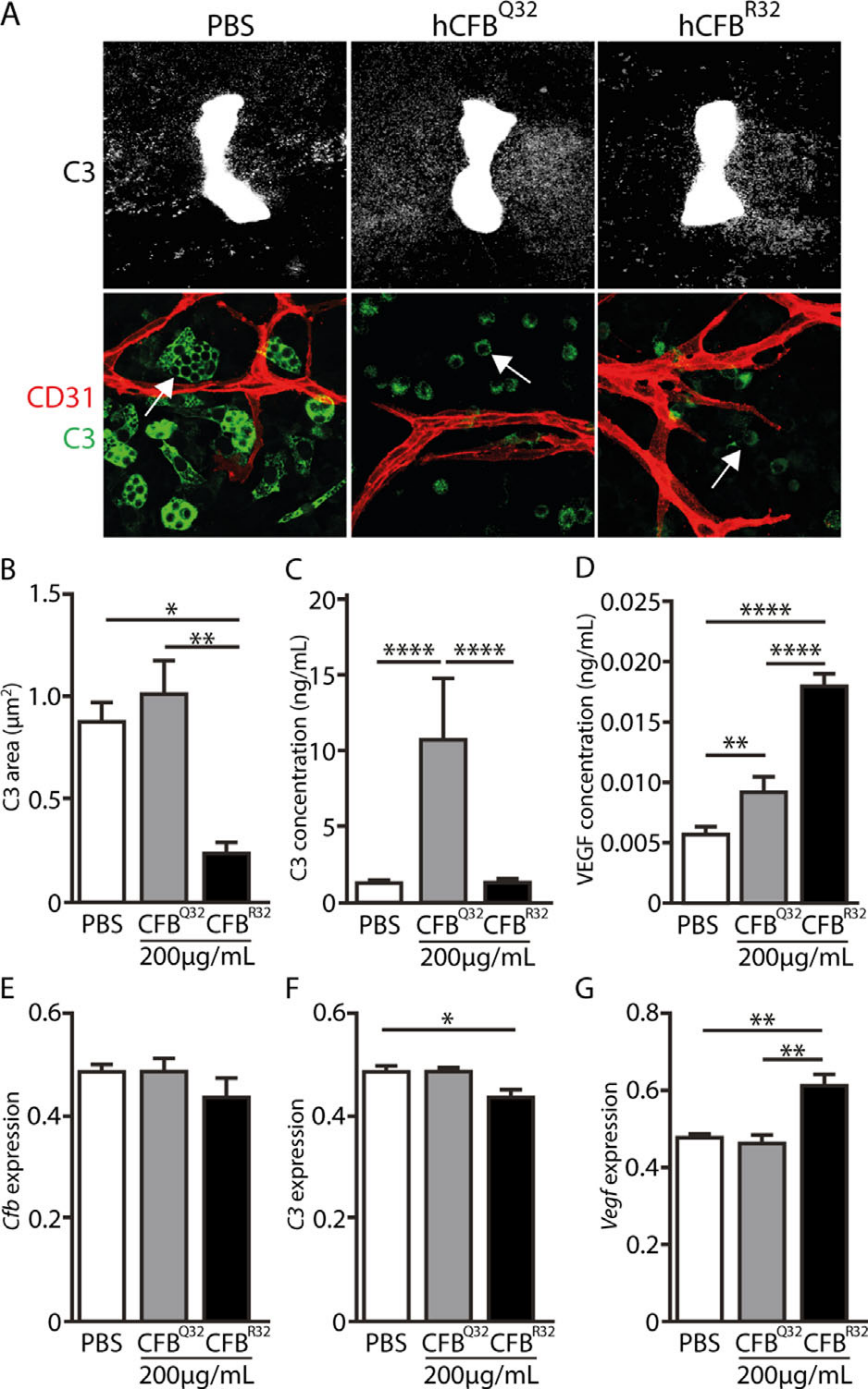
Impact of human CFB variants on C3 and VEGF. (**A**) *Upper panel*: Representative images of metatarsals treated with media supplemented with PBS, 200 µg/mL CFB^Q32^, or 200 µg/mL CFB^R32^ and stained for C3. *Scale bar*: 200 µm. *Lower panel*: Representative maximum-intensity projection confocal images of metatarsals treated as described above and stained for CD31 (*red*) and C3 (*green*). The *white arrow* in the PBS treatment indicates a vacuole in a C3-positive cell and the *white arrows* in the CFB^Q32^ and CFB^R32^ treatments indicate small, round C3-positive cells without vacuoles. *Scale bar*: 20 µm. (**B**) The area of C3 staining (µm^2^) was quantified from low-power images. (**C**) Quantification of C3 protein by ELISA in conditioned media from metatarsals treated with PBS, 200 µg/mL CFB^Q32^ and CFB^R32^. (**D**) VEGF levels were measured by ELISA in conditioned media from metatarsals treated as described above, with data normalized to PBS. (**E–G**) Metatarsal cultures were treated as indicated for 9 days, and 24 hours after the last treatment, transcript expression levels of *Cfb*, *C3*, and *Vegf* respectively were determined by quantitative PCR. Expression values were normalized to the housekeeping gene β-tubulin and to PBS. *Error bar*s: SEM. *P < 0.05, **P ≤ 0.01, ****P ≤ 0.001.

CFB activation results in the catalytic conversion of C3 to C3b and the generation of the proenzyme C3bB, and previous studies have reported that CFB^Q32^ is inferior to CFB^R32^ in driving this conversion. We therefore sought to identify C3 in the metatarsal model system and to determine its abundance in relation to the presence of the CFB variants. To do this, we maintained metatarsals in the presence of CFB^Q32^ and CFB^R32^ and determined the abundance of C3/C3b signal by immunocytochemical staining using a C3 antibody that is specific for both inactive and active (C3b, iC3b, C3c) forms of C3 (Fig. 3A). For the two CFB variants, the area of C3/C3b staining was quantified in low-magnification images, revealing significantly less C3/C3b staining after CFB^R32^ treatment compared to PBS or CFB^Q32^ (Fig. 3B). In line with this and previous observations,^6^ we showed by ELISA that secreted C3/C3b was increased in the conditioned media of CFB^Q32^-treated metatarsals compared to PBS control and CFB^R32^ (Fig. 3C), consistent with the activity profiles of the two CFB variants. To test whether CFB^R32^ was stimulating blood vessel growth in a VEGF-dependent manner, we quantified VEGF levels by ELISA in the conditioned media in metatarsals treated with the two CFB variants. This analysis indeed revealed a significant difference in the level of VEGF between the control and the two CFB variants and between the variants themselves (Fig. 3D), suggesting that angiogenesis driven by CFB^R32^ may occur, at least in part, via a VEGF-dependent mechanism and that CFB^Q32^ is less active in this model. To assess if there was correlation between protein and mRNA levels, we analyzed gene expression of *Cfb*, *C3*, and *Vegf* in the metatarsal cultures treated with PBS, 200 µg/mL CFB^Q32^ and CFB^R32^. *Cfb* transcripts appeared similar across treatments (Fig. 3E). Interestingly, *C3* mRNA levels (Fig. 3F) did not closely correlate with C3 protein levels (Fig. 3C), suggesting that the CFB variants have little impact on C3 expression themselves and that C3 protein levels are instead a reflection of the different activities of the two variants. Additionally, *Vegf* transcripts were found to be markedly upregulated following CFB^R32^ compared to CFB^Q32^ treatment (Fig. 3G), in line with the increased VEGFA concentration in conditioned media and increased angiogenesis with CFB^R32^.

### CFB and Macrophages

**Figure 4.**
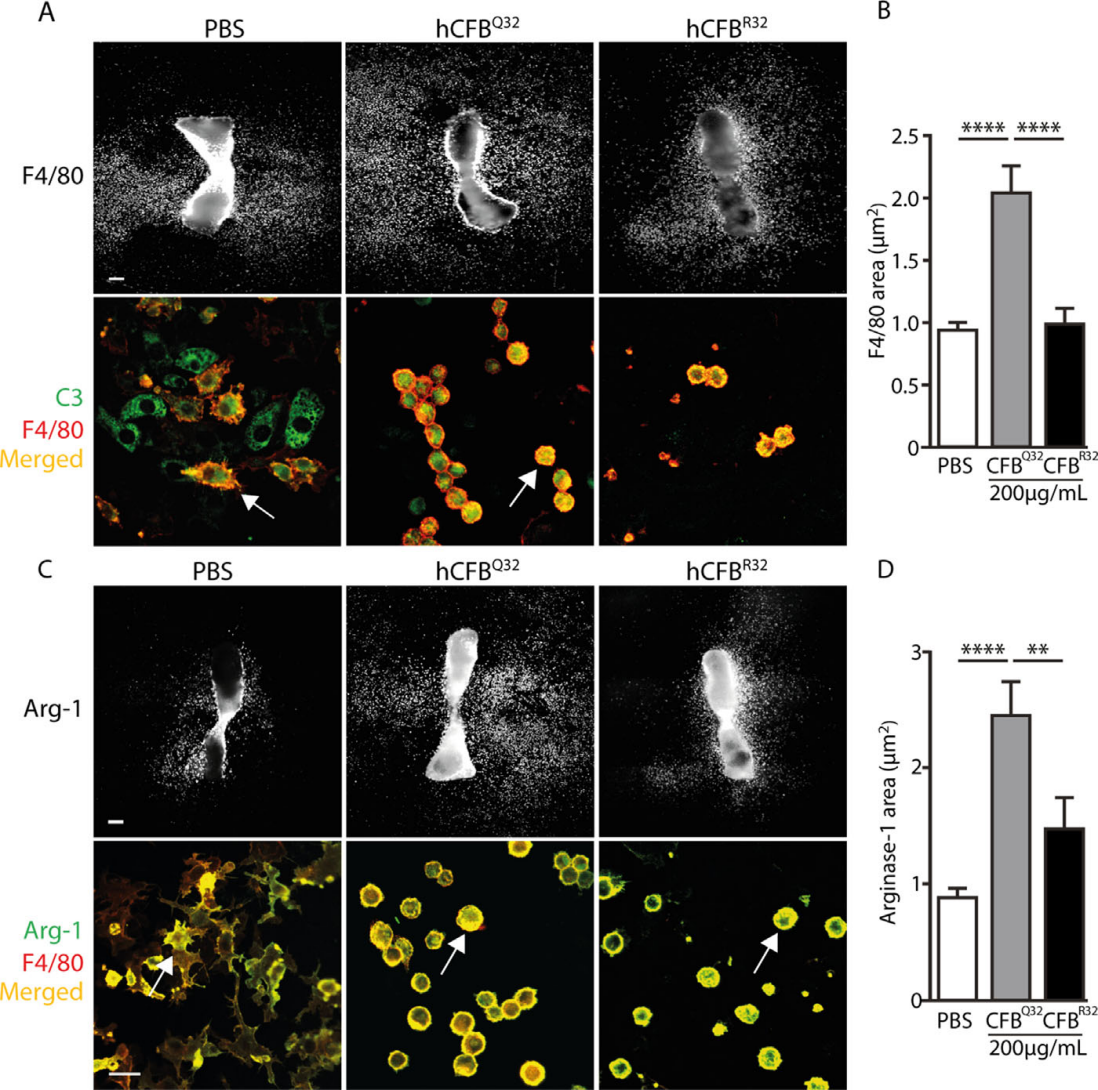
Impact of human CFB variants on macrophages. (**A**) *Upper panel*: Representative images of metatarsals treated with PBS, 200 µg/mL CFB^Q32^, or 200 µg/mL CFB^R32^ and stained for F4/80. *Scale bar*: 200 µm. *Lower panel*: Representative 40× maximum-intensity projection confocal images of metatarsals treated as described above and stained for F4/80 (*red*) and C3 (*green*). Cells that are double stained for both markers appear *yellow* (*white arrows*). In the PBS treatment, the double-positive cells are elongated and ramified, while in the CFB-treated metatarsals, they are round. *Scale bar*: 20 µm. (**B**) The area of F4/80 staining (µm^2^) is quantified from 4× images and normalized to PBS. (**C**) *Upper* *panel*: Representative images of metatarsals treated with PBS, 200 µg/mL CFB^Q32^, or 200 µg/mL CFB^R32^ and stained for arginase 1. *Scale bar*: 200 µm. *Lower panel*: Representative 40× maximum-intensity projection confocal images of metatarsals treated as described above and stained for F4/80 (*red*) and arginase 1 (arg-1) (*green*). Cells that are double stained for both markers appear *yellow* (*white arrows*). *Scale bar*: 20 µm. (**D**) The area of arginase 1 staining (µm^2^) is quantified from 4× images and normalized to PBS. *Error bars*: SEM. **P ≤ 0.01, **** *P* ≤ 0.001.

Since macrophages are responsive to C3/C3b and can either promote or inhibit angiogenesis depending on the microenvironment, we next examined their density and phenotype in metatarsals treated with the wild-type CFB^R32^ and the less active variant CFB^Q32^. Macrophages and monocytes were identified by immunostaining in metatarsals treated with PBS, 200 µg/mL CFB^R32^, or 200 µg/mL CFB^Q32^ (Fig. 4A, upper panel). Confocal images of metatarsals treated as described above revealed some C3/C3b-positive cells that were also F4/80 positive (white arrows in Figure 4A, lower panel). The double-stained cells acquired different phenotypes depending upon the treatment: control cells were generally more elongated with irregular perimeter consistent with these being in a resting state and typically appeared in groups, whereas CFB-treated cells were smaller with a regular round shape, indicative of activation, and more evenly distributed. The area of F4/80 staining was quantified revealing that upon CFB^Q32^ treatment, there was a significantly higher density of F4/80-positive cells compared to control and CFB^R32^-treated metatarsals (Fig. 4B). Additionally, the colocalization of C3/C3b and F4/80 and vice versa was evident in approximately 50% of cells across treatments, demonstrating that only half of the two cell populations were positive for both markers. However, the amount of F4/80 staining overlapping C3/C3b was significantly greater with hCFB^Q32^ compared to hCFB^R32^ (data not shown).

There are two main subtypes of macrophages: classically activated M1 macrophages, which are proinflammatory and antiangiogenic, and alternatively activated M2 macrophages, which are anti-inflammatory and proangiogenic.^16^ However, the distinction between the two types is neither absolute nor binary, and therefore the need to more accurately identify macrophage subtypes is important because of their distinct effects on angiogenesis and inflammation. Nevertheless, the distribution of the M2 macrophage marker, arginase 1, as assessed by immunocytochemistry, revealed an increase in total staining area, indicating that there were significantly more arginase 1–positive cells after CFB^Q32^ treatment compared to metatarsals treated with PBS or CFB^R32^ (Fig. 4D).

## Discussion

CFB is a key positive mediator in the alternative pathway of complement activation where it drives the formation of the C3 convertase. Its absence or loss of function would therefore be predicted to lead to complement dysregulation. An imbalance in the proteins of the alternative pathway of the complement system is not only involved in immune and inflammatory diseases such as AMD^17^^,^^18^ but also other conditions such as atherosclerosis and cancer, as well as physiologic processes such as angiogenesis and lipid metabolism.^19^^–^^22^ Investigating the role of CFB in angiogenesis and inflammation is of interest since SNPs in this gene have been identified as being genetically associated with reduced risk (CFB^W32^ and CFB^Q32^) in developing AMD.^4^^–^^7^ Since it is only the human variants of CFB that are known to be associated with ocular pathology and to enhance the relevance of our studies to human disease, we generated variants of human CFB as recombinant proteins rather than the orthologous mouse proteins.

Our primary objective was to find out whether CFB has a role in the formation of new blood vessels, in light of previous studies linking CFB to pathologic neovascularization in mouse models, and its association with neovascular AMD. We first addressed the hypothesis that a mixture of complement proteins, rather than a single complement factor, is proangiogenic in the mouse metatarsal model. In these experiments, we used normal human serum, which contains thermo-labile components such as CFB, C5b-9, CFI, C2, C8, C7, and the thermo-stable complement C3.^23^^,^^24^ In the presence of 10% heat-inactivated human serum, we observed a significant reduction in angiogenic sprouting and branching in the metatarsal assay, showing that complement factors may contribute to the angiogenic process.

To specifically focus on the possible role(s) of CFB as a factor that contributes to angiogenesis in the metatarsal assay, we generated CFB variants as recombinant proteins and observed that the addition of exogenous CFB^R32^ at physiologic levels increased angiogenesis compared to the protective variants CFB^Q32^ and CFB^W32^. This suggests that increased vessel outgrowth and branching are indeed caused by complement activation since CFB^R32^ is the most active of the three variants. This is consistent with the observation of decreased membrane-bound C3/C3b in CFB^R32^-treated metatarsals and the accumulation of C3/C3b in the conditioned media from metatarsals after CFB^Q32^ treatment. C3 and C3b proteins are both present in the metatarsal assay media and potentially synthesized and expressed by several cells types, including endothelial cells, macrophages, and fibroblasts.^25^ Reduced C3/C3b staining is most likely the result of C3 being broken down by the C3 convertase into C3a and C3b fragments, of which the latter drives a positive feedback loop, leading to amplification of complement activation. In support of a role for endothelial cells in the production of C3, Human Umbilical Vein Endothelial Cells (HUVECs) in culture have been reported to secrete C3 and also CFB, albeit in low amounts during basal conditions,^26^ and in previous work, we observed induction of CFB expression in the pathologic retinal blood vessels of several mouse models.^27^ Our observations here are in line with previous biophysical studies that revealed that CFB^R32^ binds with highest affinity to C3b, and as such, it catalyzes the most effective C3 convertase formation and the most efficient C3 breakdown.^6^

In this context, it was interesting to note that VEGFA protein levels in the conditioned media and *Vegf* transcripts of CFB^R32^-treated metatarsals were significantly increased, suggesting that this variant, apparently acting at the transcriptional level, stimulates angiogenesis via a VEGFA-dependent mechanism. Whether the elevated VEGFA levels were a consequence of increased C3 convertase formation or reflect another undescribed activity of CFB is not clear. Nevertheless, our observations fit with the known role of VEGF in neovascular AMD and also its involvement in tumor angiogenesis and in the interplay between the immune system, inflammatory cells, and cancer cells.^28^ As macrophages may be an important source of VEGF,^29^^,^^30^ we also investigated their phenotype in the metatarsal assay. Multiple macrophage subpopulations exist, and their identification can shed light on their role in pathology. In our study, addition of CFB to mouse metatarsals led to macrophage activation, at least on the basis of a phenotypic switch in which the shape typically became round compared to the ramified shape of macrophages in the control samples,^31^ although similar changes in macrophage cell morphology may also be due to de-differentiation.^32^ Whether the macrophage phenotype shift is a direct or indirect effect of CFB is unknown. There is evidence that CFB and C3 can be expressed by macrophages, and thus *Cfb* is upregulated in mouse macrophages via Toll-like receptor type 4 activation.^33^ Interestingly, we also found an increased number of F4/80-positive macrophages in mouse metatarsals in response to CFB^Q32^, which may have been the result of increased cell proliferation.

An important distinction in macrophage phenotype and function is the separation into M1 and M2 subtypes. It is well established that a spectrum of phenotypes means that this is not always a simple binary issue. Here we observed that most F4/80-positive macrophages in CFB^Q32^-treated metatarsals were at the M2 end of the phenotype spectrum due to the increased arginase 1 staining. These macrophages are typically alternatively activated, anti-inflammatory, and proangiogenic.^16^^,^^34^ However, despite promoting an increase in the proangiogenic M2 population, CFB^Q32^ stimulated significantly less angiogenesis than CFB^R32^. These observations, together with the correlation between CFB^R32^ and higher levels of VEGFA, indicate that the effects of CFB on angiogenesis are not solely mediated by macrophages.

In summary, in this study, we have provided evidence that CFB^R32^, CFB^Q32^, and CFB^W32^ have distinct biological activities with regard to angiogenesis, with the more common CFB^R32^ driving increased vessel growth accompanied by elevated levels of VEGFA.
